# Heidenhain variant of Creutzfeldt-Jakob disease: An autopsy study from India

**DOI:** 10.4103/0972-2327.48856

**Published:** 2009

**Authors:** Monica Kher, Medha Y. Rao, P. T. Acharya, Anita Mahadevan, S. K. Shankar

**Affiliations:** Department of Medicine, MS Ramaiah Medical College and Hospital, MSRIT Nagar, Bangalore - 560 054, India; 1 Department of Neurology, MS Ramaiah Medical College and Hospital, MSRIT Nagar, Bangalore - 560 054, India; 2 Department of Neuropathology, National Institute of Mental Health and Neurosciences, Bangalore - 560 029, India

**Keywords:** Creutzfeldt-Jakob disease, MR imaging, immunostaining, prion protein (PrP^sc^)

## Abstract

Prion diseases are rare, progressive and fatal neurodegenerative diseases characterized by long incubation period and short clinical course. We present a rare case of Heidenhain variant of Creutzfeldt-Jakob disease, occurring in a 55-year-old lady presenting with dementia, cortical blindness, and myoclonic jerks. She succumbed to the disease within 8 weeks of onset of symptoms. MRI revealed hyperintense signals on T2WI and fluid attenuated inversion recovery (FLAIR) images in basal ganglia and fronto-temporal and parietal cortex, sparing thalamus, striate cortex and globus pallidum. Abundant abnormal prion protein deposits (PrP^sc^) were detected in caudate, putamen, thalamus, cingulate and striate cortex, in comparison to frontal and parietal cortex while no deposits were found in globus pallidum. MRI changes did not correlate with degree of spongy change, gliosis or prion protein deposition. The cause for abnormal signal changes in MRI and FLAIR images remains unclear.

## Introduction

Transmissible spongiform encephalopathies are a complex group of rare but fatal neurodegenerative disorders encompassing multiple clinical syndromes. The disorders are caused by the accumulation of post-translationally modified, insoluble, fibrillary prion protein (PrP^sc^) in the brain causing spongiform (vacuolar) degeneration of neuropil in the brain and variable amyloid plaque formation. The global annual incidence of CJD ranges from 0.3 to 1.1 per million population,[1] while in India an incidence of 0.085 per million (8.5 cases in more than one billion population) is recorded by the CJD Registry at Department of Neuropathology, National Institute of Mental Health and Neurosciences, Bangalore (unpublished data). Based on Master's criteria,[2] 52% were categorized as “definite” when there was histopathological confirmation and the remaining as “probable” with rapidly progressive dementia, myoclonus and extrapyramidal symptoms in the absence of histological evidence. We report a case of Heidenhain variant of sporadic CJD in a 55-year-old lady presenting with cognitive deficits, cortical blindness and extrapyramidal symptoms.

## Case Report

A 55-year-old lady who had vegetarian habits presented with dizziness of three months duration with intermittent vomiting. She had no history of fever or headache. Two weeks prior to admission, her relatives noticed behavioral changes in the form of irritability and occasional agitation. She was confused with irrelevant speech. She complained of blurring of vision and required help for activities of daily living. She was hospitalized on 16 November 2006. At admission, she was disoriented with loss of memory for recent events. She had poor vision in both eyes with normally reactive pupils suggestive of cortical blindness. Menace reflexes were absent and optic fundi were normal. She had rigidity in all four limbs with normal deep tendon reflexes and flexor plantar response. No history of immunization, hormone substitution therapy, or surgical procedures was recorded in the past. Over the next 5 weeks of hospitalization, the patient's neurological state rapidly deteriorated with worsening of cognitive function and increasing rigidity of limbs and trunk. She developed myoclonic jerks terminally.

Routine hematological and biochemical profile including thyroid functions and vitamin B12 levels were normal. CSF analysis revealed protein of 40 mg/dl, glucose of 100 mg/dl and only 2 cells/cmm (lymphocytes). CSF was negative for HSV antibodies. Bacterial and mycobacterial cultures were sterile. Serological tests for systemic vasculitis were negative, and she was seronegative for HIV and VDRL. Tests for 14-3-3 in CSF and molecular genetic studies for substitution in PRNP gene could not be carried out due to lack of facility in the country and logistic problems in transfer of material to a foreign laboratory.

**Figure 1:**
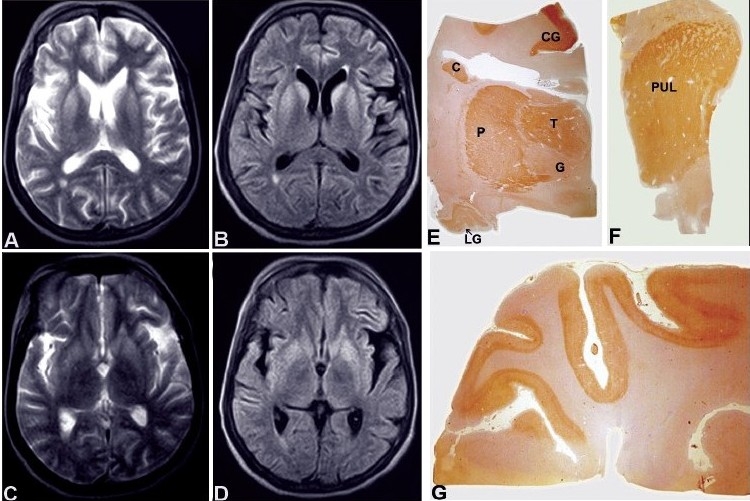
Axial T2-weighted (A, C) and FLAIR images (B, D) show bilateral hyperintense signals in caudate nucleus and putamen. Prion protein (PrP^sc^) immunostaining on whole mount preparation in coronal plane (E, F) shows strong labeling of caudate nucleus, putamen, medical thalamic nuclei and pulvinar but mild staining in globus pallidum. Note: labeling of lateral geniculate body (LG) and cortical ribbon of cingulate gyrus (CG). Whole mount of striate cortex (G) shows strong diffuse immunolabeling of cortical ribbon for (PrP^sc^). [E, F, G – Prion protein immunostain (KG9 monoclonal antibody) ×1.5]

Cranial CT scan was reported normal. T1-weighed MRI showed sulcal prominence. The T2-weighted fast spin echo sequences revealed abnormal high signal intensities bilaterally in corpus striatum [Figure 1A,C]. FLAIR sequences showed increased signal in the deep cortical layers of temporal lobe extending to perisylvian region, bilateral parasagittal frontal, parietal and occipital cortex, caudate nucleus and putamen [Figure 1B,D]. The globus pallidum and thalamus were normal. ADC values could not be calculated for technical reasons. A clinical diagnosis of CJD was entertained on the basis of rapidly progressive dementia, cortical blindness, extra pyramidal features, myoclonus and MR imaging features. Periodic sharp wave discharges were found on EEG which was strongly suggestive of CJD, while visual evoked potentials (VEP) were normal bilaterally.

She deteriorated rapidly in neurological status, became bedridden and succumbed to the illness within 5 weeks of onset of illness.

Informed consent for partial autopsy confined to removal of the brain was obtained. The brain was fixed in 10% formalin for three weeks prior to sectioning. Tissue blocks from different anatomical areas were obtained and immersed in 96% formic acid for 24 h to reduce infectivity. Tissues were embedded in paraffin, and large blocks were sectioned at 6 μ, stained with haematoxylineosin and luxol fast blue for myelin. In addition, sections through frontal, temporal cortex and striate cortex, basal ganglia, thalamus, cerebellum and different levels of brainstem were immunostained with monoclonal antibodies to Glial Fibrillary Acidic Protein (GFAP) to label glial elements and KG-9 to detect abnormal prion protein (PrP^sc^) (antibody specific domain lying between aminoacid residues 140 and 180 of prion protein-BBSRC Resource Centre: Courtesy Dr. J.W. Ironside, National Surveillance Unit, Western Hospital, Edinburgh, UK).

**Figure 2:**
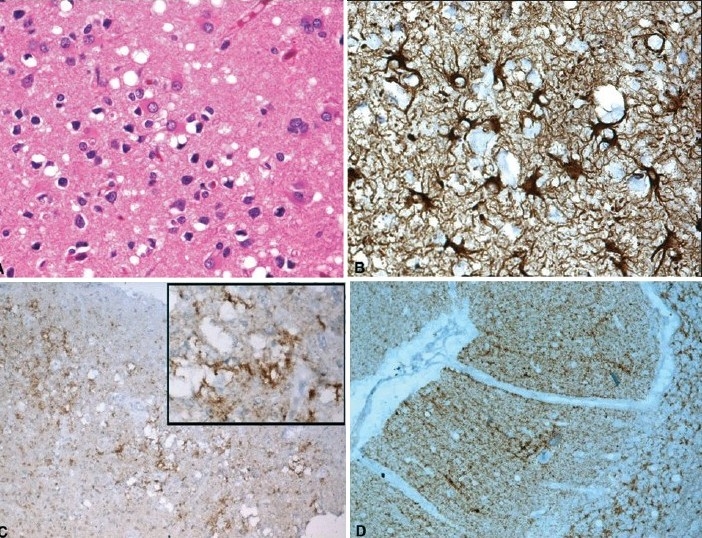
A. Spongy change, reactive astrocytosis and neuronal loss in frontal cortex. [H&E X200]. B. GFAP immunostaining highlights glial processes encircling spongy spaces. [GFAP Immunoperoxidase X360]. C. Perivacuolar labeling in superficial layers of frontal cortex. Inset highlights perivacuolar pattern of prion protein deposition. D. Synaptic pattern of immunostaining of the cerebellar folial molecular layer, highlighting the linear deposition along dendrites and the internal granular layer. Immunoperoxidase – KG9 monoclonal antibody to prion protein. [C ×100; D ×160; Inset ×280]

The brain revealed diffuse cortical and cerebellar folial atrophy. The striate cortex in the occipital lobe, frontal cortex [Figure 2A], limbic structures including cingulate gyrus, entorrhinal cortex, and parahippocampal gyrus showed variable spongy change, neuronal depletion and astrocytosis. In the diencephalic zones, body of the caudate nucleus, dorsomedial and ventrolateral thalamic nuclei, pulvinar of thalamus showed asymmetric spongy change, with islands of neuronal loss and reactive astrocytosis with similar changes in molecular layer of cerebellum. GFAP immunostaining highlighted reactive and hypertrophic astrocytes, the cell processes encircling the spongy vacuoles [Figure 2B]. Immunolabeling with antibody to prion protein revealed granular synaptic pattern of prion protein deposition in the limbic cortex, occipital striate cortex (visual cortex) [Figure 1G], caudate nucleus, thalamus [Figure 1E,F], lateral geniculate body and nuclear zones in the mid brain. In addition, perivacuolar labeling was observed in the superficial layers of striate cortex [Figure 2C], and frontal cortex, sparing the hippocampus and parahippocampal areas. The cerebellar molecular layer had synaptic pattern of granular deposits of (PrP^sc^) highlighting the linear dendritic branches without prion plaque formation [Figure 2D].

On correlating the MRI T2W-FLAIR signal changes with histomorphological changes, the hyperintense signal changes in striatum and cortex did not correspond to degree of gliosis. Although dense PrP^sc^ deposits was noted in caudate nucleus and putamen corresponding to hyperintense signal, the thalamus including pulvinar had dense PrP staining without associated hyperintense signals, while globus pallidum that had focal deposits also did not show hyperintensities. In this case, clinical presentation with cortical blindness, rapidly progressive dementia, myoclonus and extrapyramidal symptoms with histological evidence of spongiform encephalopathy with prion protein deposition in striate cortex, lateral geniculate body in addition to diencephalic nuclei is characteristic of Heidenhain variant of CJD.

## Discussion

The clinical features that suggest the diagnosis of CJD is often preceded by a prodromal period in which nonspecific clinical signs occur, which include fatigue, sleep and memory disturbances, behavioral changes, vertigo and ataxia as noted in the present case. The most characteristic clinical features are rapidly progressive dementia, myoclonus, motor disturbances (extrapyramidal, cerebellar, pyramidal and anterior horn cell) and EEG revealing periodic short wave bursts. A definitive diagnosis of CJD rests on the demonstration of neuropathological triad of neuronal loss, spongiform change and reactive astrocytosis in the absence of inflammatory reaction. Immunostaining with antibody to PrP^sc^ either as diffuse granular synaptic pattern, perivacuolar deposits in spongy areas or as immunolabeled amyloid plaques is diagnostic. However, these pathological changes vary considerably from case to case. Masters and Richardson[2] reviewing the clinical spectrum of human prion disease subclassified CJD into Heidenhain variant with visual symptoms and severe occipital pathology, striatal variant resembling Huntington's disease, thalamic variant including familial fatal insomnia, cerebellar variants resembling Kuru, clinical forms with oculomotor disturbances resembling progressive supranuclear palsy and panencephalitic form with white-matter demyelination. No difference in localization of prion protein (PrP^sc^) in brain in these clinical variants of CJD is described.

By electron microscopy, the neuropil spongy change is noted to manifest as swelling of neuritic processes and presynaptic axonal terminals with loss of internal organelles and accumulation of lacy membrane profiles. The degree of reactive astrocytosis correlates with extent of neuronal loss.

The earlier studies though failed to recognize significant signal changes on MRI,[3] Gertz *et al*.[4] described hyperintensities on T2-weighted images in basal ganglia in a case of CJD. Subsequent publications demonstrated bilaterally symmetric, diffuse hyperintensities in basal ganglia in cases of CJD[5,6] Zeidler *et al.*,[7] in a case of new variant of CJD (nvCJD), described the pulvinar sign (hyperintensities in the posterior thalamus) that is considered to be diagnostic. Diffusion-weighted MRI, a noninvasive imaging modality to image molecular water proton diffusion occurring in the micro-environment of neuropil and fluid inversion recovery (FLAIR) sequences are found to show similar signal changes in cases of CJD corresponding to T2-weighted hyperintense signal changes and the spongiform degeneration in the neuropil with microvacuolation of neuritic processes.[8,9] The MRI changes are considered to precede the disease even before the diagnostic abnormalities are noted on EEG or in protein 14-3-3 values.[8] In addition, the high signals in T2-weighted sequences have been linked to astrogliosis.[6] Haik[10] *et al*. reported the deposition of pathological PrP^sc^ in regions with increased signal changes, which was evident even in brain structures with normal signals. However, spongiform change and gliosis in different brain regions showed no clear association with high intensity signal changes in MRI.[10,11] In the present case, caudate nucleus and putamen with hyperintense signals on T2W and FLAIR images showed heavy deposition of PrP^sc^. On the other hand, thalamus including pulvinar with deposits of prion protein failed to show signal changes, while globus pallidum with no signal changes had minimal PrP staining. Similarly, the frontal and temporal cortex with high signals had diffuse, focal and variable (PrP^sc)^ deposits, whereas striate cortex with dense immunolabeling failed to show signal changes. The reason for the lack of association of PrP^sc^ deposition with MRI and DWI changes is still not clear and does not appear to bear a direct correlation with the pathological substrate. Haik[10] *et al.* suggested that accumulation of the hydrophobic amyloid isoforms of (PrP^sc)^ could contribute to modifications of T2W, DWI and FLAIR images in the brain. The diagnostic role of various imaging modalities of MRI in cases of spongiform encephalopathies needs further evaluation. This can at best be used as an adjunct with strong clinical suspicion.
